# Benefits in pain perception, ability function and health-related quality of life in patients with failed back surgery syndrome undergoing spinal cord stimulation in a clinical practice setting

**DOI:** 10.1186/s12955-018-0887-x

**Published:** 2018-04-19

**Authors:** Luciana Scalone, Furio Zucco, Angelo Lavano, Amedeo Costantini, Marisa De Rose, Paolo Poli, Gianpaolo Fortini, Laura Demartini, Enrico De Simone, Valentino Menardo, Mario Meglio, Paolo Cozzolino, Paolo A. Cortesi, Lorenzo G. Mantovani

**Affiliations:** 1 Research Centre on Public Health (CESP), University of Milano Bicocca, Via Cadore 48, I-20900 Monza, Italy; 2 CHARTA Foundation, Milan, Italy; 3 Azienda Ospedaliera Salvini, Garbagnate Milanese, Italy; 4 Università Magna Grecia, Catanzaro, Italy; 5 Ospedale Clinicizzato Ss.Annunziata, Chieti, Italy; 6 Azienda Ospedaliera Universitaria Pisana, Pisa, Italy; 7 Azienda Ospedaliero Universitaria Ospedale di Circolo e Fondazione Macchi, Varese, Italy; 8 IRCCS Fondazione Salvatore Maugeri, Pavia, Italy; 9 A.O.R.N. “S.G. Moscati”, Avellino, Italy; 10 Azienda Ospedaliera Santa Croce e Carle di Cuneo, Cuneo, Italy; 11 Policlinico Universitario Agostino Gemelli, Rome, Italy

**Keywords:** Failed back surgery syndrome, Spinal cord stimulation, Health-related quality-of-life, Pain intensity, Disability

## Abstract

**Background:**

Failed back surgery syndrome (FBSS) represents one main cause of chronic neuropathic or mixed pain, functional disability and reduced Health Related Quality of Life (HRQoL). Spinal Cord Stimulation (SCS) can be a value for money option to treat patients refractory to conventional medical management (CMM).

We estimated from real-world data: 1) the amount of reduced levels of HRQoL of target patients compared to general population, 2) the relationship between pain intensity, functional disability, and overall HRQoL, and 3) the improvement of patients’ health from SCS intervention, and 4) we give some insights and make some suggestions on the selection of a battery of patients’ reported health instruments for use in routine clinical practice.

**Methods:**

At recruitment (before SCS) and every 6 months for 2 years after SCS a battery of questionnaires/tests were completed: the generic EQ-5D and SF-36 for HRQoL, the specific Numerical Rating Scale (NRS) to measure pain intensity, and Oswestry Disability Index (ODI) to measure functional disability. We conducted multilevel regression analyses to investigate the association of HRQoL with the NRS and ODI indexes; multiple regression analyses to compare EQ-5D data with those of the general population adjusted for age, sex and education, and statistical tests to compare the changes of HRQoL, NRS and ODI estimates at baseline with those measured during the follow-up.

**Results:**

Eighty patients (40% male, mean age = 58 years) participated. HRQoL was significantly worse in the patients than in the corresponding general population. Pain, functional disability and HRQoL significantly related each other during follow-up, Significant improvements (*p* < 0.001) in pain intensity, functional capability and HRQoL were reached after 6 months from SCS and generally remained stable during follow-up. Specific instruments provided detailed information on disability and pain, while generic instruments assessed the overall HRQoL and allowed a comparison with the general population’s one.

**Conclusions:**

SCS + CMM treatment reaches a statistically significant and probably a clinically relevant improvement in pain perception, functional disability and HRQoL in patients with FBSS refractory to CMM. An appropriate selection of instruments for use in clinical practice is crucial for a routine assessment of health perception in patients, aimed to guide decisions for optimal treatment.

## Background

Failed Back Surgery Syndrome (FBSS) is a relatively common condition causing chronic low back and/or leg pain persisting or recurring after one or more lumbar surgeries, associated with functional disability, low levels of Quality of Life [1, 2], and high rate of loss of productivity, with a significant economic impact [3, 4]. It has been estimated that affects 0.61% of general population, with an annual incidence of 0.033% [3]. It has been reported that 30% of patients having lumbar spinal surgery will develop FBSS [2]. Low levels of Health-Related Quality-of-Life (HRQoL) have been reported for patients with severe chronic pain [5]. A systematic review reports that HRQoL in patients with FBSS is lower than in patients with other chronic conditions such as neuropathic pain disorder (e.g. diabetic polyneuropathy) and other conditions like stroke or heart failure [6]. The impact of FBSS and its management on individuals’ health and its economic cost to society are considerable [4]. In patients who experience persistent pain after conventional medical management, Spinal Cord Stimulation (SCS) might be recommended [7]. More recently, specific recommendations for appropriate SCS implantation have been published [8]. In 2008 the National Institute for Health and Care Excellence (NICE) recommended the use of SCS for the treatment of neuropathic pain, including those caused by FBSS, and underlined the need of observational research able to generate robust evidence about the durability of benefits of SCS in the real world context [9]. Among the studies conducted in this area in the past years [10–14], the PRECISE study is the first real-world study showing the value for money of SCS in patients with FBSS refractory to conventional medical management. In particular, the cost-utility acceptability curve obtained from the analyses of the data suggests that under the assumption that decision makers’ willingness to pay per Quality-Adjusted-Life-Years (QALYs) is €60,000, SCS implantation is cost-effective in 80 and 85% of cases, according to the NHS’s and societal point of views, respectively.

Further investigations were conducted to assess the relationships and the trend of patients’ health after treatment with SCS [13]. However, no real world data were analysed in those studies, despite the suggestions by the NICE [15]. The present work had more aims: to estimate 1) the amount of reduced levels of HRQoL that patients with FBSS can have compared to the corresponding general population, 2) the relationship between pain intensity, functional disability, and overall HRQoL, and 3) the improvement of patients’ health in 2 years from SCS intervention in a clinical practice context. Furthermore, 4) we give some insights and make some suggestions on the choice of the patients’ reported health instruments, which can contribute to perform a routine complete health assessment aimed to optimize treatment benefits in clinical practice.

## Methods

### Subjects and setting

The dimension of the sample in the PRECISE study was decided adopting a combination of statistical and pragmatic approach, according to previous scientific experience focusing on patients with similar characteristics (e.g. [10]), and on the resources available for the conduction of the present study in a naturalistic context. Considering the specificity of the FBSS with refractoriness to conventional medical management, which requires high level of expertise necessary for the adoption of SCS, we found 9 specialized centers (6 pain units and 3 neurosurgery wards) able to participate across Italy, all having at least five years of experience in the management of patients with FBSS treated with SCS. Each center could potentially contribute with around 8–10 eligible patients assigned to receive SCS following clinical practice. Accordingly, we expected to reach around 72–90 patients, which was higher than the dimension reached from a single country in other studies previously conducted, including the PROCESS Randomized Clinical Trial [10]. In the PROCESS trial [10], which main objective was to assess clinical effectiveness of SCS in patients with FBSS, the sample size was established to include 50 patients per parallel arm from different countries, under the hypothesis that 30% percent of the patients improved at least 50% on the self-reported pain scale (primary outcome) within the first 6 months from treatment initiation. Applying this hypothesis in our study for the same primary outcome measured with the NRS scale, considering its different design based on a pre- post-treatment single arm or participants, a sample size of 80 subjects would provide estimates at a power > 85% with an alpha< 5%.

Between June 2005 and October 2007, all consecutive patients who satisfied the eligibility criteria were invited and accepted to participate. Inclusion and exclusion criteria used for this study, specified in the Additional file 1, reflect recommendations that have been recently published [16, 17].

Eligible patients received information on: 1) the aim of the study, 2) the SCS surgical procedures and potential clinical outcomes, 3) the technical variables (e.g. the self regulation parameters), related both to the external pulse generator (EPG) during the stimulation test period (STP) and to the totally implantable pulse generator (IPG); 4) the possible complications; 5) the data collection procedure. Eligible patients had to sign an informed consent form after receiving all the necessary information on the aim of the study, the type of data and the method of data collection. The study participation of each center was previously approved by the Local Ethics Committee, present in each hospital according to the Italian regulations on clinical research.

### Procedure

At the enrollment the participants underwent a percutaneous lead implantation adopting standardized clinical practice [2, 8, 18] and following the study protocol homogeneously by all the participating centres. The procedure was carried out under local anesthesia with patient in prone position. A Tuohy needle was inserted 2 cm lateral to the midline obliquely into the posterior epidural space and a lead was introduced and advanced upward under fluoroscopic control. When the appropriate lead level was reached in order to maximize paresthesia coverage of painful area, a percutaneous extension wire was implanted and connected to an external stimulator. The patients were observed during a Stimulation Test Period (STP), with a minimum duration of 15 days. Those who responded positively to the STP were implanted under local anesthesia with non-rechargeable IPG, placed in a subcutaneous pocket at the level of the abdominal wall, and were followed up to 24 months. The test screening was considered positive when patients experienced at least 50% pain relief and at least 80% overlap of pain with stimulation-induced. We did not continue to collect data on patients that did not respond positively to the STP and on patients that for different reasons stopped the study before the scheduled 24-month follow-up period.

### Observational period

**Fig. 1:**
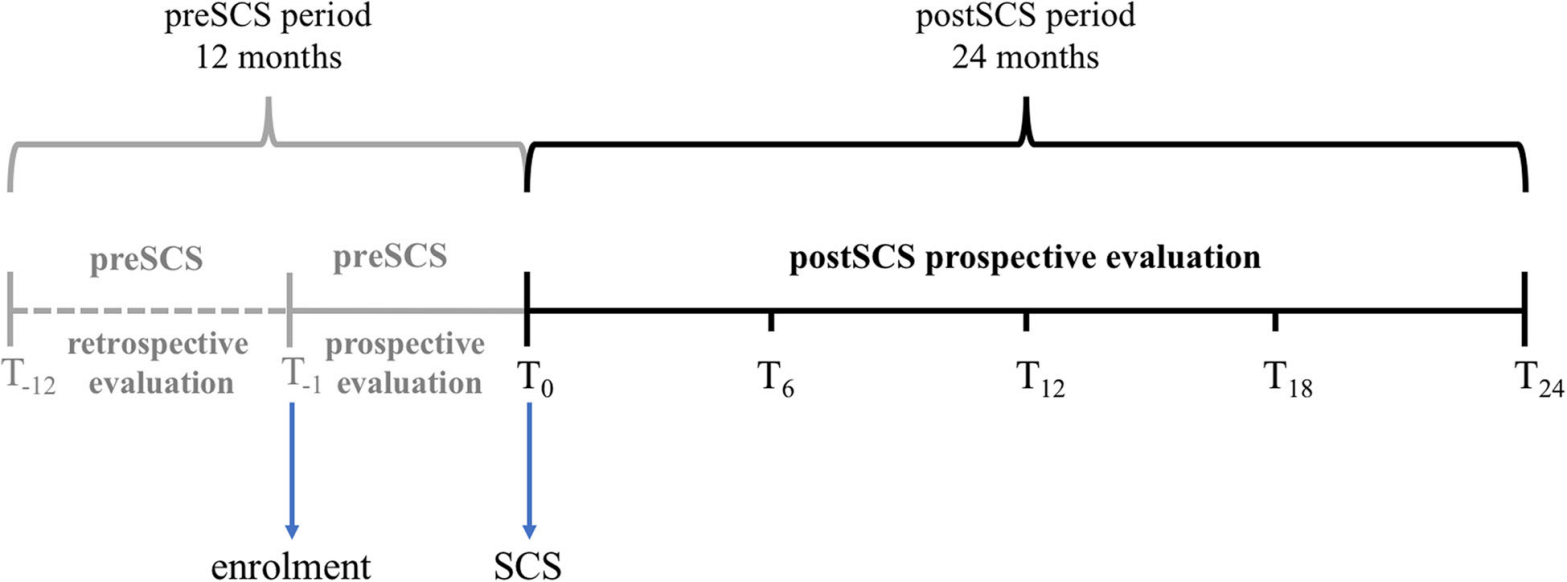
Schematic definition and duration of the observational period. Schematic representation of the observational period. All the patients were enrolled at T_− 1_, completed the questionnaires about the previous 12 months, and underwent a simulation test period (STP), named preSCS period. At T_0_, those who responded positively to the STP were implanted with implantable pulse generator (postSCS period) and completed again the questionnaires at that time and at T_6,_ T_12,_ T_18_ and T_24_

A schematic definition and duration of the observational period is reported in Fig. 1. Namely, the observational period included a preSCS and a postSCS period. The preSCS period included the 12-months period before SCS: 11 months before enrolment and 1 month from enrollment to SCS intervention. The postSCS period was intended to be up to 24 months after the SCS intervention and was divided in 6-months periods according to the scheduled follow-up visits.

### Data and data collection

Data were collected using a Case Report Form (CRF) and a patient diary. Together with information on resources consumption used to conduct the economic evaluation [13] we collected data on socio-demographic and clinical characteristics, pain intensity, physical ability and HRQoL. Data were collected on the 12 months before enrolment (preSCS period) and on the postSCS period at each scheduled 6-month follow-up visit.

Socio-demographic data included age, gender, marital status, working status, and education collected by the physician at enrolment.

Pain intensity was recorded using the Numerical Rating Scale (NRS), which is scored from 0 (no pain) to 10 (the most intense pain imaginable) [19]. In particular, each patient was asked to score on the NRS the mean and the maximum level of pain perceived at the low back and one or both the legs, in the previous 12 months (at enrolment) and in the previous 6 months (during follow-up).

The level of disability was measured with the Oswestry Disability Questionnaire, which is a back-specific questionnaire covering 10 dimensions of functional ability: intensity of pain, lifting, ability to care for oneself, ability to walk, ability to sit, sexual function, ability to stand, social life, sleep quality, and ability to travel. Each patient was asked to assign to each domain one out of six possible levels of severity from 0 (no limitation) to five (greatest possible limitation) according his/her current perception. The percentage of disability, corresponding to the total ODI score, is obtained adding up the scores, dividing the total by 50 and multiplying the result by 100. A score up to 20% indicates minimal disability, 21–40% indicates moderate disability, 41–60% indicates severe disability, 61–80% indicates level of pain that interfere with all aspects of the patient’s life requiring intervention, 81–100% indicates patients who are bed bound [20].

As regards HRQoL, the patients self-completed a battery of 2 generic questionnaires: the Medical Outcome Study Short Form 36 (SF36) [21] and the EQ-5D [22]. These questionnaires were chosen for their capability to assess both physical and psychological components of health, as they allow comparing health within and between different clinical conditions and with the general population. Interestingly, these instruments have been used also in other recent studies on FBSS [5, 6, 23]. SF-36 assesses HRQoL using 35 questions referring to the previous month grouped in eight dimensions scoring between 0 (corresponding to worst possible state) and 100 (corresponding to best possible state). Namely, physical functioning, role-physical, and bodily pain are more related to the physical component of health; social functioning, role-emotional, and mental health are more related to the mental component of health; finally, energy/vitality and general health relate to both components [21, 24]. The eight domains can be grouped into two summary scores, one specific for physical health (Physical Component Summary - PCS) and the other for mental health (Mental Component Summary – MCS) [25].

The EQ-5D was developed to describe value individuals’ health. The tool consists of two parts: a descriptive system (EQ-5D profile) consisting of 5 domains, namely “mobility”, “self-care”, “anxiety/depression”, “usual activities” and “pain/discomfort”. In the EQ-5D-3 L version, the descriptive system includes three levels of severity per domain (“no problem”, “some/moderate problems”, “extreme problems/impossible to do”). The second part of the questionnaire consists of a visual analogue scale (EQ-5D VAS) measuring the overall HRQoL, ranging from 0 (worst imaginable health state) to 100 (best imaginable health state). With the EQ-5D the respondents are asked about their HRQoL on the current day. The responses of the EQ-5D descriptive system can be converted into utility indexes by means of an algorithm that uses population-based (social) values. The utility index corresponds to the estimate of value of health with a score anchored between 0, corresponding to death, and 1, corresponding to perfect health. Utility indexes are widely used in different disease areas and recommended for the calculation of QALYs to be applied in economic evaluations of health technologies [9].

### Data analyses

Patients’ demographic, clinical characteristics at baseline and their health state at enrollment and during follow-up were described using absolute and/or relative frequencies for the categorical variables, while continuous variables such as age and some health status measures (e.g. VAS, utility), were summarized by mean values along with standard deviation (±SD) as dispersion measures.

To proceed with the analyses, we decided to adopt the Last Observation Carried Forward (LOCF) approach, aimed at avoiding possible bias in favor of the SCS treatment (e.g. if only patients that benefited from this procedure remained in the study) due to missing follow-up data of patients who did not continue the study due to STP failure (hence they likely had no improvement), or for other reasons during the observational period, were managed using the [26]. In particular, for each patient that did not continue the study until the scheduled end of observational period, we carried forward to 24 months the NRS, ODI, SF-36 and EQ-5D information available from the last data available. On the database managed with the LOCF approach we applied all the following analyses.

Using the EQ-5D descriptive system we calculated utility scores by means of an algorithm that uses population-based (social) values estimated in Italy [27].

To reach objective 1, i.e. to compare HRQoL of the patients with that of the corresponding general population, we used the reference EQ-5D-3 L data for the Italian general population [28]. Since the HRQoL data available from the general population were not longitudinal but cross sectional, to compare them with the longitudinal data from the patients we assumed that HRQoL in the general population is overall constant. We adopted multiple regression analyses, including either the EQ-5D VAS or utility index as dependent continuous variables, and age, sex and education as potential confounders (independent variables) widely recognized in the scientific literature (e.g. [28]). In particular, we applied 5 regression models for each dependent variable, one per time period, each one including the same HRQoL data from the general population. The predictor of interest, to distinguish between general population (reference) and patients, was introduced as a binary explanatory variable. The other independent variables were included to adjust the results for age (linear variable), sex and education (categorical variables). According to the distribution of the data, we applied the linear regression analysis to the VAS, while to the utility index we applied the Tobit model, which accounts for ceiling effect [29, 30].

To estimate the relationship between pain intensity, functional disability, and HRQoL during the overall observational period (objective 2) we adopted multilevel random intercept regression linear analyses, known also as “hierarchical model”. With these models we estimated the adjusted association between HRQoL with NRS and ODI across the time, i.e. taking into account for the repeated measures per participant, with level one of the hierarchy being observations over time within a patient and level two being the patient. Multilevel models have been used to analyse longitudinal HRQoL data [31–33]. In particular we performed 4 regression models. In each model we included one among the following as dependent continuous variables: EQ-VAS, utility index, SF-36 PCS and MCS scores. In every model we included mean NRS and ODI index as independent continuous variables of interest to find the association with HRQoL, while age baseline HRQoL, time of data collection (continuous variables), sex, education, and previous surgery (categorical variables) were all included as potential confounders. Again, we chose these independent variables according to our large scientific and clinical experience in the field of the Outcomes Research and FBSS.

To compare HRQoL scores and percentages for the different time periods (objective 3), we calculated the changes that each patient had between two subsequent time periods (i.e. 6 months compared to recruitment, 12 months compared to 6 months, 18 months compared to 12 months and 24 months compared to 18 months). Then, we calculated and report the mean of the individual changes among all the patients and performed the related parametric (paired Student’s) or non-parametric (paired Wilcoxon signed rank) statistical tests, depending on type (continuous or ordinal) and distribution of data, which was assessed for normality using with the Shapiro-Wilk test. Since we conducted multiple comparisons, we adopted the Bonferroni correction to test for the differences between each pair of tests [34].

For all tests, *P*-values < 0.05 (two-tailed tests) were considered statistically significant and are reported together with and 95% confidence intervals where appropriate.

All analyses were conducted using Stata SE 12 (Stata Corp, Texas, US) software.

## Results

A total of 80 valid patients were recruited in the study. Description of patients’ socio-demographic and clinical characteristics at baseline are specified in Table 1. After a mean of 46 days from enrolment, the patients were implanted with a lead and observed during the STP. Eight patients (10%) had a negative test response. After an average of 30 days from lead implantation, patients with a positive test response were implanted with IPG. During follow-up the IPG was replaced in 8 patients and the lead was replaced twice in 1 patient, for dislocation. As regards the occurrence, reason and amount of missing data, during follow-up 17 patients stopped to be observed because: 1 died for stroke and 1 died for infarction, which are common events among people aged on average 58 years, 5 had adverse events, 7 were lost to follow-up for unknown reasons, and 2 had lost therapeutic effect, while 1 withdrew the consent to participate. The total number of participants were 67 at 6 months, 62 at 12 months, 58 at 18 and 55 at 24 months of follow-up.

**Table 1:** Patients’ characteristics at study enrolment time

Description of characteristics	Mean(±SD) or frequency
Total number of patients	80
Age (mean ± SD years)	58 (±13)
Male (%)	32 (40%)
Education, (%)	
Primary	39 (49%)
Lower secondary	27 (34%)
Upper secondary	12 (15%)
Graduate	1 (1%)
None	1 (1%)
Number of previous surgical interventions, n (%)	
1	23 (33%)
2	31 (44%)
3	13 (19%)
4	3 (4%)
Information not available	10
Age (mean ± SD years) at pain onset	48 (±14)
Time (mean ± SD years) between pain onset and recruitment	11 (±9)

At baseline the patients reported on average high levels of pain perception, low levels of ability function and general HRQoL (Table 2). The mean and the maximum pain levels measured with the NRS were 7.6 and 9.2, respectively. High levels of pain were assessed also with the SF-36 (bodily pain mean score = 21.2), with the EQ-5D, showing that 65% of the patients reported extreme pain or discomfort, and with the ODI, which pain intensity levels 4 or 5 were reported by 37.5% of the patients. Among the other aspects assessed with the ODI, we found that 47 to 70% of the patients reported maximum levels of disability in standing, sexual function, social life, travelling and lifting, while 21 to 32% of the patients had serious problem in sleeping, sitting and personal care. Consequently, the total ODI score was high, with a mean value of 61.6. Similarly, physical functioning, role-physical and consequently the overall PCS of the SF-36 had the lowest mean scores (23.3, 22.4 and 26.7, respectively), while 41.3% of the patients reported inability to do usual activities with the EQ-5D. Accordingly, the mean utility score and EQ VAS were relatively low, i.e. 0.421 and 37.4, respectively.

**Table 2:** Pain perception, disability and HRQoL changes during follow-up

Domains and indexes		Baseline	∆ 6 m–0 m*	∆ 12 m–6 m*	∆ 18 m–12 m*	∆ 24 m–18 m*
NRS	maximum score (mean ± SD)	9.2(±0.1)	-2.5^c^	0.3	−0.3	0.0
	mean score (mean ± SD)	7.5(±0.2)	−2.6^c^	0.2	−0.4	0.3
ODI	Pain intensity, (levels 4–5)	37.5%	−24.8^c^	2.5	−2.4	1.3
	Personal care, (levels 4–5)	25.6%	−15.5^a^	2.5	−1.1	− 1.3
	lifting, (levels 4–5)	69.6%	−5.1	−1.3	14.8^a^	− 5.1
	ability to walk, (levels 4–5)	32.5%	− 3.4	− 12.7 ^b^	4.1	1.3
	ability to sit, (levels 4–5)	25.3%	−8.9	−6.3	0.1	2.6
	ability to stand, (levels 4–5)	46.8%	−17.7^a^	− 1.3	6.8	3.8
	sleep quality, (levels 4–5)	21.3%	−8.6^a^	− 1.3	1.4	0.0
	sexual function, (levels 4–5)	49.3%	−10.1	−6.8	− 0.9	0.0
	social life, (levels 4–5)	57.5%	−27.1^c^	−2.5	−4.8	−1.3
	ability to travel, (levels 4–5)	60.0%	−25.8^c^	−1.3	− 0.9	2.6
	total score (mean ± SD)	61.6 (±15.0)	−16.0^c^	− 0.1	−2.5	− 0.6
SF-36	physical functioning (mean ± SD)	23.2 (±15.8)	14.3^c^	0.4	−0.5	−2.6^a^
	role-physical (mean ± SD)	22.4 (±33.1)	11.4^a^	1.1	−1.1	−4.3
	bodily pain (mean ± SD)	21.2 (±14.1)	21.4^c^	−0.6	0.0	−0.6
	social functioning (mean ± SD)	31.3 (±18.8)	18.2^c^	−1.4	2.4	−1.9
	role-emotional (mean ± SD)	29.4 (±36.5)	14.8^a^	− 2.7	6.1	− 4.7
	mental health (mean ± SD)	42.8 (±19.8)	8.0^b^	−1.0	2.7	−3.4^a^
	energy/vitality (mean ± SD)	30.5 (±17.5)	10.8^c^	−2.7	1.8	−0.5
	general health (mean ± SD)	33.2 (±13.3)	3.8^a^	0.4	1.3	− 2.0
	PCS (mean ± SD)	26.7 (±6.2)	5.8^c^	0.3	−0.7	−0.6
	MCS (mean ± SD)	35.8 (±9.9)	4.9^b^	−1.3	2.2	−1.4
EQ-5D	Mobility, (extreme problems)	16.3%	−5.0	−2.5	−1.3	0.0
	Self-care, (extreme problems)	10.0%	−3.8	1.3	−3.8	0.0
	Usual activities, (extreme problems)	41.3%	−20.0^b^	2.5	−3.8	1.3
	Pain/Discomfort, (extreme problems)	65.0%	−37.5^c^	0.0	0.0	1.3
	Anxiety/Depression, (extreme problems)	25.0%	−1.3	−3.8	−2.5	0.0
	Utility index (mean ± SD)	0.421 ± 0.303	0.174^c^	0.021	0.025	−0.011
	VAS (mean ± SD)	37.4 ± 2.4	18.6^c^	−1.0	3.3	−0.9

^a^*p* < 0.05, ^b^*p* < 0.01, ^c^*p* < 0.001. *P*-values were corrected with Bonferroni’s method

^*^On NRS, ODI and EQ-5D profile domains, a negative change (**∆**) indicates improvement, a positive change indicates worsening, on SF-36 single and summary domains, on the EQ-5D utility index and VAS a positive change (**∆**) indicates improvement, a negative change indicates worsening

The patients had a significant impaired HRQoL compared to the Italian general population of the same age, sex and education (Table 3): the estimated EQ VAS and the utility scores at baseline were on average 37.392 and 0.506 less, respectively, among the patients than in the general population with the same characteristics specified.

**Table 3:** Comparison of HRQoL between patients and general population

	Baseline			6 months f-up			12 months f-up			18 months f-up			24 months f-up		
EQ-VAS	*Regr. coef.*	*95% CI*	*P*	*Regr. coef.*	*95% CI*	*P*	*Regr. coef.*	*95% CI*	*P*	*Regr. coef.*	*95% CI*	*P*	*Regr. coef.*	*95% CI*	*P*
Age (years)	−0.337	− 0.361,-0.313	< 0.0001	− 0.336	− 0.361,-0.312	< 0.0001	− 0.337	− 0.361,-0.312	< 0.0001	− 0.337	− 0.362,-0.313	< 0.0001	− 0.337	− 0.361,-0.312	< 0.0001
Sex: Male (reference)	–	–	–	–	–	–	–	–	–	–	–	–	–	–	–
Female	−1.714	−2.528,-0.901	< 0.0001	− 1.699	− 2.513,-0.885	< 0.0001	− 1.664	− 2.478,-0.849	< 0.0001	− 1.620	−2.436,-0.805	< 0.0001	−1.639	− 2.454,-0.825	< 0.0001
Subjects: General population (ref.)	–	–	–	–	–	–	–	–	–	–	–	–	–	–	–
Patients	−37.392	−41.356,-33.428	< 0.0001	−18.337	−22.266,-14.408	< 0.0001	−20.694	− 24.626,-16.762	< 0.0001	− 17.054	− 21.017,-13.090	< 0.0001	− 17.568	−21.528,-13.608	< 0.0001
Education: none (reference)	–	–	–	–	–	–	–	–	–	–	–	–	–	–	–
Primary/Middle school	3.693	−2.621,10.007	0.252	4.358	−1.879,10.595	0.171	2.026	−4.215,8.267	0.525	2.774	−3.478,9.025	0.384	4.411	−1.835,10.657	0.166
Upper school/University	7.788	1.495,14.080	0.015	8.407	2.191,14.623	0.008	6.111	−0.109,12.332	0.054	6.792	0.561,13.023	0.033	8.440	2.215,14.665	0.008
Intercept	91.777	85.257,98.297	< 0.0001	91.080	84.635,97.525	< 0.0001	93.354	86.904,99.803	< 0.0001	92.620	86.159,99.081	< 0.0001	90.985	84.529,97.440	< 0.0001
EQ-Utility	*Regr. coef.*	*95% CI*	*P*	*Regr. coef.*	*95% CI*	*P*	*Regr. coef.*	*95% CI*	*P*	*Regr. coef.*	*95% CI*	*P*	*Regr. coef.*	*95% CI*	*P*
Age (years)	−0.003	−0.003,−0.003	< 0.0001	− 0.003	− 0.003,− 0.003	< 0.0001	−0.003	− 0.003,− 0.003	< 0.0001	−0.003	-0.003,-0.003	< 0.0001	-0.003	-0.003,-0.003	< 0.0001
Sex: Male (reference)	–	–	–	–	–	–	–	–	–	–	–	–	–	–	–
Female	−0.052	− 0.060,-0.044	< 0.0001	− 0.052	− 0.060,-0.044	< 0.0001	− 0.052	−0.060,-0.043	< 0.0001	− 0.051	− 0.060-0.043	< 0.0001	− 0.052	−0.060-0.044	< 0.0001
Subjects: general population (ref.)	–	–	–	–	–	–	–	–	–	–	–	–	–	–	–
Patients	−0.506	−0.545,-0.467	< 0.0001	− 0.306	− 0.345,-0.267	< 0.0001	− 0.294	−0.333,-0.254	< 0.0001	− 0.255	− 0.294,-0.216	< 0.0001	− 0.268	−0.307,-0.229	< 0.0001
Education: none (reference)	–	–	–	–	–	–	–	–	–	–	–	–	–	–	–
Primary/Middle school	0.005	−0.059,0.069	0.879	−0.055	−0.119,0.009	0.093	−0.089	− 0.154,-0.024	0.007	− 0.070	−0.134,-0.005	0.034	−0.060	− 0.125,0.004	0.065
Upper school/University	0.035	−0.029,0.098	0.284	−0.028	−0.092,0.037	0.401	−0.062	− 0.127,0.003	0.062	− 0.042	− 0.106,0.022	0.200	−0.033	− 0.097,0.031	0.312
Intercept	1.179	1.113,1.245	< 0.0001	1.241	1.174,1.308	< 0.0001	1.275	1.207,1.342	< 0.0001	1.253	1.186,1.320	< 0.0001	1.246	1.179,1.312	< 0.0001

Note: the column on the left lists the independent variables introduced in the regression models for the VAS and for the EQ-Utility index. The other columns show the regression coefficients, the 95% confidence intervals and the p values estimated for each independent variable analysed with the dependent variables EQ-VAS or EQ-utility index. Positive regression coefficients indicate a direct association and negative regression coefficients indicate an inverse association between the dependent variables and the independent variables. 95% Confidence intervals and *p* values indicate the level of significance of the regression coefficients

**Fig. 2:**
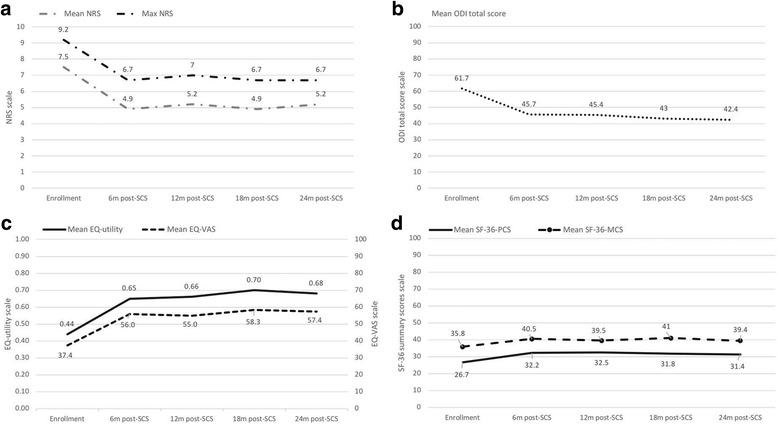
Trends of main outcome measures. Trends during 24 months of follow up, i.e. at enrollment, at 6 m, 12 m, 18 m and 24 m post-SCS, of mean and maximum pain score measured with NRS (**a**), of disability score measured with ODI (**b**), of HRQoL assessed with EQ-utility index and VAS (**c**) and with the SF-36 PCS and MCS scores (**d**)

The results of the multilevel regression model (Table 4) show that the during the full observational period, on average patients with higher levels of pain (NRS) and of disability (ODI) had worse levels of HRQoL (EQ-5D VAS and utility index, SF-36 PCS and MCS scores), on adjusting for the possible confounders specified in the table. This negative association was statistical significant in every parameter. Figure 2 shows the trends during follow up of main scores measuring pain (a), disability (b) and HRQoL assessed with the EQ-5D [5] and SE-36 (d). Generally, the curves similarly decreased (pain and disability) or increased (HRQoL) steeper during the first 6 months and slower in the following period, although this trend was lighter for SF-PCS and SF-MCS scores. To see detailed results about this trend Table 2 shows the changes in each domain between 2 time periods (6 months versus enrolment, 12 versus 6 months, 18 versus 12 months and 24 versus 18 months). In particular, the mean or percentage changes estimated for the time between enrollment and the following 6 months, corresponds always to an improvement after the intervention, as shown by the signs of the differences in each domain or index, which improvement is often statistically significant. Furthermore, some changes estimated 6 months after the SCS intervention probably reach also a minimum clinically important difference (MCID): the authors of a recent study [35], report that in patients with FBSS, the estimated MCID was 2.2 and 2.7 for the low back and the leg NRS, respectively (we estimated a mean change of 2.6, which could include pain perception in both areas), 9 for the ODI total score (we estimated a mean change of 16), 10.2 and 4.0 for the SF-36 PCS and MCS (we obtained 5.8 for the PCS, and 4.9 for MCS). As regards the EQ-5D utility index, of which we estimated an average difference of 0.174, a MCID of approximately 0.08 [36] and 0.17 [37] has been proposed for the treatment of low back pain.

**Table 4:** Adjusted relationship of pain (mean NRS) and functional disability (ODI) with HRQoL paramenters (EQ-utility, EQ-VAS, SF-PCS, SF-MCS) during follow-up

	*EQ-Utility*			*EQ-VAS*			*SF-PCS*			*SF-MCS*		
Independent variables	Regr. Coef.	95% CI	P	Regr. Coef.	95% CI	P	Regr. Coef.	95% CI	P	Regr. Coef.	95% CI	P
Mean NRS	−0.018	−0.027,-0.009	< 0.001	−3.187	−4.095,-1.985	< 0.001	−0.745	−1.108,-0.382	< 0.001	−1.371	−1.938,-0.805	< 0.001
ODI	−0.009	−0.010,-0.008	< 0.001	− 0.253	− 0.402,-0.112	0.001	− 0.228	−0.275,-0.181	< 0.001	− 0.083	−0.159,-0.007	0.031
Baseline HRQoL score	0.217	0.143,0.291	< 0.001	0.121	0.016,0.246	0.038	0.467	0.298,0.636	< 0.001	0.453	0.306,0.600	< 0.001
Age (years)	0.001	−0.002,0.002	0.930	−0.066	− 0.226,0.097	0.432	− 0.012	−0.092,0.067	0.761	−0.018	− 0.135,0.099	0.767
Sex												
Female – reference	–	–	–	–	–	–	–	–	–	–	–	–
Male	0.001	−0.044,0.045	0.974	−3.341	−8.314,0.941	0.159	−0.652	−2.727,1.424	0.538	−1.088	−4.045,1.869	0.471
Education												
None - reference	–	–	–	–	–	–	–	–	–	–	–	–
Lower secondary school	−0.014	−0.079,0.050	0.661	1.554	−5.639,6.915	0.642	−1.475	−4.466,1.516	0.334	−0.085	−4.395,4.224	0.969
Upper secondary school/university	−0.039	−0.087,0.010	0.124	−1.456	−6.014,3.982	0.558	0.951	−1.291,3.193	0.406	−0.175	−3.343,2.993	0.914
Previous surgery												
None or 1 - reference	–	–	–	–	–	–	–	–	–	–	–	–
More than 1	0.004	−0.044,0.052	0.861	−1.254	−6.014,3.982	0.628	0.621	−1.291,3.193	0.587	−2.326	−5.579,0.926	0.161
Visit (time)	0 .003	−0.009,0.014	0.642	1.945	0.440,3.415	0.011	−0.339	−0.714,0.097	0.077	−0.331	− 0.962,0.300	0.303
Intercept	1.039	0.904,1.174	< 0.001	80.165	65.235,93.185	< 0.001	35.095	27.878,42.313	< 0.001	35.753	26.383,45.123	< 0.001
Random intercept (SD)	0.061	0.042,0.088	< 0.0001	3.714	1.485,9.284	0.117	3.713	2.982,4.624	< 0.0001	4.995	3.887,6.418	< 0.0001
Random residuals (SD)	0.140	0.129,0.153		15.116	13.717,16.657		4.706	4.338,5.106		7.966	7.344,8.641	

The column on the left lists the independent variables introduced in the regression models, and the intercept and the random component of the model. The other columns show the regression coefficients and the standard deviations of the random intercept and random residuals, the 95% confidence intervals and the p values estimated for each independent variable analysed with the dependent variables EQ-utility index, EQ-VAS, SF-PCS and SF-MCS. Positive regression coefficients indicate a direct association, and negative regression coefficients indicate an inverse association between the dependent variables and the independent variables. Confidence intervals and *p* values indicate the level of significance of the regression coefficients

Instead, the changes estimated in the following periods of follow-up were minimal and generally not statistically significant. The improvements found among the patients are visible in the comparison with the general population (Table 4), although they did not reach the mean levels of HRQoL of the corresponding general population: the difference of the mean EQ VAS and the utility levels between patients and the general population decreased from − 37.392 and − 0.506 at baseline to − 17.568 and − 0.268 at 24 months, respectively, keeping a statistical significant difference of *p* < 0.0001.

## Discussion

With the PRECISE study we obtain in a real-world context, involving a relatively high number of patients, a detailed picture of health progression in patients with FBSS refractory to CMM and treated with SCS added to CMM. The benefits reached by the participants, combined with the economic results previously published [13], show that the SCS is an effective and valuable treatment strategy in this category of patients. Past research confirms that SCS treatment is effective in pain relief, improves HRQoL and disability in patients that are refractory to CMM [1, 5, 11, 17, 23, 38]. Furthermore, the results of the systematic literature review published few months ago by Cho et al. [39] on treatment outcomes for patients with FBSS, show that the spinal cord stimulation and the epidural adhesiolysis could be effective to control chronic pain due to FBSS, while other treatment options specified in the review show poor or inconclusive evidence. However, the authors specify that the results of the review are however not very strong, and cost-effectiveness results could help to clarify the value of the different treatment options available for FBSS. In their systematic review, [40] conclude that the cost-effectiveness of SCS is still unclear and evidence for SCS role in FBSS is controversial. To note, both Cho et al. and Warszak et al., [39, 40] exclude from their review, for not clear reasons, the results of some studies, including the cost-effective and the cost-utility analysis we published in 2015 [13], which could have influenced their conclusion [41]. In other recent literature reviews [42, 43], the authors conclude that evidence exists for SCS as a safe, effective and efficient treatment for several chronic pain conditions. Although most of the higher-quality evidence is relatively short-term, clinical experience with the durability of treatment benefit of SCS in these patients is promising.

Our results show that, at enrolment the patients had serious impairment in terms of pain perception, in functional disability and related aspects, and in HRQoL at a whole, which was significantly lower than the HRQoL assessed in the corresponding general population. During the follow-up period, pain and functional disability measured with the condition-specific instruments NRS and ODI, respectively, significantly related with HRQoL assessed with the EQ-5D and the SF-36. After only 6 months from SCS intervention, an improvement of health was found in every domain of every instrument used, which was generally statistically significant. According to the findings of past research, we can consider these improvements probably clinically relevant: in their recent study, Park et al., [35] report that in patients with FBSS, the estimated MCID was 2.2 and 2.7 for the low back and the leg NRS, respectively, which is very close to our estimated mean change of 2.6 (which includes both leg and back pain); they estimated a MCID of 9 for the ODI total score, which is even lower to our estimate of 16; furthermore, they estimated a MCID of 10.2 and 4.0 for the SF-36 PCS and MCS, respectively, while we obtained 5.8 for the PCS, and 4.9 for MCS. As regards the EQ-5D utility index, of which we estimated an average difference of 0.174, a MCID of approximately 0.08 [36] and 0.17 [37] has been proposed for the treatment of low back pain in other works. In the periods following the first 6 months from SCS, the estimated mean changes of pain, functional ability and HRQOL decreased and were generally not statistically significant. Furthermore, although the improvements are visible, the patients approached to but did not reach the mean HRQoL levels of the general population.

Our interpretation of these results is that the main advantages of the SCS intervention were reached and perceived already in the following few months, and afterwards it generally remained stable. Our results are quite similar to those of the previous PROCESS randomized clinical trial [10, 11, 23, 31, 44], which showed correlation between the different parameters of health considered, and a significant improvement already after the first month of treatment with SCS + CMM, in comparison with the effects of a control group treated with only CMM. However, while the PROCESS study reported promising results until 24 months of treatment with SCS + CMM, although based on slight improvements, our results show that in several domains, the sign of the change estimates after the first 6 months from SCS, was opposite to the expected one, but not significant, above all in the final follow-up period, from 18 to 24 months. The current unavailability of data on a follow-up period longer than 24 months makes it impossible to confirm and clarify these observations, but some possible explanations can be given. First, the LOCF approach adopted to manage the missing data from the time the patients were lost from observation (25 patients, 31% of the study sample), could bias our results in a pessimistic direction, which is a potential limitation in our study: namely, to each patients that was lost from observation, including those who did not respond positively to the STP (10%), we used the last data observed as applicable in the remaining follow-up period, assuming but not being certain that no change occurred in his/her health. Accordingly, an underestimate of improvements may be present in our results compared to those from the PROCESS clinical trial, in which only the data of the patients who remained observed for the full observational period were analysed.

A second potential limitation could be that no control group was available in this study, due to that it was performed in a real-world context. Actually, we do not have reasons to doubt about the validity of our results, since previous research demonstrated the benefits of SCS in pain relief, HRQoL and disability in patients that are refractory to CMM [1, 10, 11, 17, 23, 38, 45]. In particular, the PROCESS study [10] was a parallel-arm RCT demonstrating similar benefits of SCS in patients similar to those involved in the PRECISE study. To notice, because of the high effectiveness registered in the CMM + SCS compared to the CMM arm, for ethical reasons, after six months of follow-up the patients had the opportunity to crossover to the CMM + SCS option [11, 23, 45].

A further potential limitation could be the possible low statistical power of the regression analyses due to a small study sample, especially the multilevel analyses conducted on our data. The sample size for the present study was decided according to a clinical effectiveness parameter (pain recovery) and according to the expected availability of participants. Although we did not decide the sample size necessary for the multilevel analyses, these were conducted consistently with those applied in the PROCESS study [44], in which data of two parallel arms of 50 patients each were analysed, while in our study all the 80 participants were recruited to be treated with CMM + SCS. Furthermore, in the model we introduced independent variables that we recognized for their potential clinical relevance to find reliable results. The results obtained with the different instruments were generally consistent, as every domain of every instrument shows a greater improvement in the first six months and an apparent stable trend in the following periods. However, the type of information provided by each instrument, and probably by apparently similar domains in the different instruments (e.g. pain, ability to walk, ability to do usual activities, mental health) is different and can have different levels of responsiveness. The literature provides with several works published in the past years [46, 47], underlining the importance of considering overall HRQoL in addition to pain perception in patients with neuropathic pain, to better understand and optimize treatment decisions, hence the importance of using more instruments to assess health in these patients. In recognizing this and similarly to previous research [48–52], in the PRECISE study we used a battery of both generic (SF-36 and EQ-5D) and condition-specific (NRS and ODI) instruments. The NRS, a single index focusing on the perception of pain, and the ODI, one of most frequently used instruments to assess functional ability in the area of spine surgery [49], are generally adopted in the Italian clinical practice. From past experience in other disease areas, the SF-36 and EQ-5D used together have generally provided quite similar results [53–56]. More specifically, in spine surgery the SF-36 has been found as the third most frequently used instruments, after the pain VAS and the ODI [49]. In addition, the EQ-5D has been shown to be the most frequently used preference-based measure (hence suitable for QALY calculations to conduct economic evaluations) in the area of low back pain [48], showing good levels of validity and responsiveness in this category of individuals. Of note, in 32 out of the 37 studies included in the review by Finch and colleagues, [48], the EQ-5D was used together with other instruments, and in particular, in 16 studies it was used with the SF-36 or the SF-12. The use of more instruments to assess health in patients with chronic pain is justified and promoted by different authors. For instance, Carreon and colleagues, [50] conclude that HRQoL assessed with the EQ-5D cannot be accurately estimated from the ODI or the NRS in patient with lumbar degenerative disorders. Other researchers [51] affirm that for patients with low back pain, although the EQ-5D index can be capable of indicating clinically important changes, it can also be less responsive than instruments specific to pain measurement, because of its more limited gradation of severity and its multidimensionality. From the results of a literature review conducted through 2010, Devine et al. [52] recommend to use the following instruments for patients with chronic low back pain undergoing spine surgery: pain VAS, ODI and a short generic HRQoL instrument such as the SF-12 or the EQ-5D, to minimize both clinician’s and patient’s burden.

Despite the presence of some domains that can be considered overlapping (e.g. pain is present in all the instruments used), the instruments used in the PRECISE study cannot be considered interchangeable or in competition, rather they should be considered complementary and together useful to take a complete picture of patients’ health. On the other hand, the practical use of all these instruments in routine clinical practice can be too burdensome or even not possible, as underlined also in other disease areas [53]. Hence, we recognize the necessity of choosing an efficient battery of questionnaires, together with the clinical instruments, for a complete assessment of patients’ health. In particular, in order to be able to calculate QALYs for the conduction of cost-utility analyses to be used in Health Technology Assessment, we recommend the use of preference-based instrument, like the EQ-5D. If a higher sensitivity and precision is required to assess the general HRQoL in the target population, the recently introduced EQ-5D-5 L, which compared with the original EQ-5D-3 L contains two additional levels per domain, has shown to be a valid and well accepted instrument in the general population and in different disease areas [28, 57, 58], and could improve the reliability and the level of information obtained with the data collected.

## Conclusion

The PRECISE study is novel in providing insights on the benefits patients can achieve in a real-world uncontrolled setting. The results obtained suggest that treatment with SCS + CMM of patients with FBSS refractory to CMM and characteristics similar to those who participated in this study, can provide clinically relevant improvement in terms of pain perception, functional ability and HRQoL as a whole. In practice, the present results, together with those on the value for money of SCS [13] can help decision makers to arrive at more informed and appropriate decisions aimed to optimize the management of FBSS patients not responding to CMM. In addition, a routine collection of HRQoL data and related parameters can be very useful to conduct informed and appropriate decisions on treatment. For this reason, the selection of an efficient combination of both generic and condition-specific instruments is crucial to obtain complete information without a too burdensome data collection process. In particular, we suggest the NRS, the ODI and the EQ-5D as an appropriate combination of instruments to be used to assess health in patients like those involved in the PRECISE study.

## Additional file

Additional file 1: Eligibility criteria of the participants. (DOCX 85 kb)

## Abbreviations

CMM: Conventional medical management
CRF: Case report form
EPG: External pulse generator
FBSS: Failed back surgery syndrome
HRQoL: Health-related quality of life
IPG: Implantable pulse generator
LOCF: Last observation carried forward
MCID: Minimum clinically important difference
MCS: Mental summary score
NICE: National Institute for Health and Care Excellence
NRS: Numerical rating scale
ODI: Oswestry disability index
PCS: Physical summary score
QALYs: Quality-adjusted life-years
SCS: Spinal cord stimulation
SF36: Short form 36
STP: Stimulation test period
VAS: Visual analog scale
