# Supplementary material for: Benefits in pain perception, ability function and health-related quality of life in patients with failed back surgery syndrome undergoing spinal cord stimulation in a clinical practice setting
Source: Health Qual Life Outcomes. 2018 Apr 19;16:68. doi: 10.1186/s12955-018-0887-x (PMC5909225; doi:10.1186/s12955-018-0887-x)
Supplement: Supplementary file 1 [file 12955_2018_887_MOESM1_ESM.docx]

Inclusion criteria

Suffering from FBSS pain that radiates to lower limbs, mono or bilaterally

Pain intensity >5 NRS 0-10 for at least 6 months, measured by the NRS 0 to 10

Being older than 18 years

Not satisfactory response to previous conservative therapy: physical and functional therapies, ≥2 pain-killer drugs, surgery

Being capable and willing to undergo the study visits

Being willing to participate in the study through a signed informed consent

Capability to manage the devices both during the test phase and after the implantation of the neurostimulator

Exclusion criteria

Predominance of pain in the lumbar region

Having other pain condition potentially interfering with the assessment of results of the target treatment (e.g., cancer pain)

Expected inability to manage the totally implanted neurostimulator and to compile the patient diary

Contraindications in receiving the SCS system (e.g., coagulation problems)

Lupuserythematosus, metabolic or other peripheral neuropathy, rheumatoid arthritis, active neoplasia

Not being a native Italian speaker

Being illiterate

Life expectancy less than 2 years

Current or planned pregnancy

Evidence of major psychiatric disorder or another personal condition known to affect the perception of pain, the treatment compliance or the assessment of the treatment effectiveness
